# Intimate Partner Aggression Committed by Prison Inmates with Psychopathic Profile

**DOI:** 10.3390/ijerph18105141

**Published:** 2021-05-12

**Authors:** José Gómez, Rosario Ortega-Ruiz, Miguel Clemente, José A. Casas

**Affiliations:** 1 Department of Psychology, University of Cordoba, 14071 Córdoba, Spain; z42goalj@uco.es (J.G.); ed1orrur@uco.es (R.O.-R.); 2 Department of Psychology, Universidade da Coruna, 15071 Coruna, Spain; miguel.clemente@udc.es

**Keywords:** intimate partner aggression, psychopathy, prison inmates, condemned, gender-based violence

## Abstract

Psychopathy and intimate partner aggression (IPA) are two concepts that usually appear concomitantly. Male violence toward women is often considered a psychopathic trait that sometimes involves the woman’s homicide by her partner and, at other times, attempted homicide. This phenomenon has been studied by conducting interviews following Hare’s model with 92 men incarcerated under a compliance regime in a Spanish prison (Córdoba). The results detected six explanatory factors of IPA as a result of attempted homicide or homicide: criminal past and delinquency, impulsivity, the need to stand out from others, lack of empathy, manipulation of others, and instability in partner relationships. The first two factors predict a occurrence of high scores on Hare’s Psychopathy Checklist. The results are discussed, and future lines of research are presented, especially focused on the concept of dehumanization and revenge.

## 1. Introduction

This work combines two problems that have not often been considered together. On the one hand, psychopathy, which in recent research, has taken on a different nuance, depending on whether it refers to its primary nature (which has been investigated mainly by [1,2,3] or its secondary nature (which has been investigated under the initial label of the dark triad, subsequently the dark tetrad, and lately, the dark personality; [4,5]. On the other hand, intimate partner aggression (IPA), which is both a social problem and a public health problem, with some authors even specifying that this is a widespread international public health crisis: [6], as some studies [7] point out that aggressions occur in 60% to 90% of relationships. This work seeks to determine the explanatory variables of partner assault carried out by a sample of inmates convicted, among other crimes, for assaulting their intimate partners.

### 1.1. Review and Meta-Analysis Articles

Several systematic reviews and meta-analytical works have either addressed the connection between psychopathy and IPA or the two aspects separately. One of the initial works Hilton and Harris [8], emphasized that the predictor variables of the assault of a female partner by the male were antisocial behavior in general, psychopathy, substance abuse, prior assault history, and previous psychological abuse. Therefore, in 2005, psychopathy was already highlighted as a risk factor for IPA.

Subsequently, Whittington et al. reviewed the instruments predicting the risk of being an aggressor that were adapted for use by mental health services and the criminal justice system [9]. They analyzed 19 databases, compiling 959 behavioral studies, published between 2002 and 2008. Again, psychopathy was considered an obvious factor.

The meta-analysis carried out was more specific of forensic populations [10], investigating the prevalence of deviant or disruptive personality traits, and analyzing 39 studies (the sample-sum of all of them was 11716 people) through self-reports. They found that forensic samples scored higher in anger, aggression, hostility, antisocial traits, psychopathy, or impulsivity than control samples, and that forensic samples show a very marked phenomenon of social desirability.

We highlight two reviews specifically relating psychopathy with IPA [6,11]. The former, selecting 41 investigations, determined that psychopathy is the most robust predictor of the perpetration of male-to-female IPA. The second study is more comprehensive, as it analyzes 43 papers, coming to similar conclusions and adding that some studies have verified associations between child abuse, psychopathy, and IPA.

Other reviews have focused on more specific aspects, relating psychopathy with alcohol consumption [12], or linking the dark personality and online antisocial behaviors [13]. As they have not referred so centrally to the relationship between psychopathy and IPA, they will not be commented on in this summary, but only briefly further on.

### 1.2. Distinctions and Points

Before reviewing the literature more precisely, we note that some of the works focus on subjects diagnosed with psychopathy who were incarcerated after being convicted of attempted murder, others were convicted of homicide, others for both types of crimes [14], whereas other studies were performed with samples of students and the general population, usually focused on the dark personality [15,16,17,18]. This study will include two types of subsample, that of prisoners diagnosed with psychopathy who have murdered their intimate partner, and those also diagnosed with psychopathy and convicted of the attempted murder of their partner. We will also refer to the debate about the sex of the aggressor and the victim.

Although all the works point out that psychopathy mostly manifests in males, who are usually the aggressors in IPA, some female psychopaths also assault males in situations of IPA. Thus, specify that female offenders are less likely to have a criminal record, are convicted to a lesser extent, are rarely convicted and imprisoned, and commit homicides with an idiosyncrasy because the victim is usually a member of their family, especially their children [14]. Moreover, according to these authors, although in males, the prediction of psychopathy as a very reliable indicator for diagnosing an antisocial personality disorder is almost completely fulfilled, this does not occur in women, as they tend to present other personality disorders.

Also, in women, the type of aggression is different. Thus, a work carried out with students from Sweden and the United Kingdom, measuring the four variables of the so-called dark tetrad (Machiavelism, subclinical narcissism, subclinical psychopathy, and subclinical sadism) and linking those scores to IPA, found a significant difference depending on sex: women were more verbally aggressive, whereas males used both verbal and physical aggression against their partners [18]. Whereas this study used both heterosexual and homosexual couples, further analyzed the composition of the couple (hetero or homosexual), the type of psychopathy (primary or secondary), and related them to the type of belief about the couple relationship [19]. The data indicated that the aggressor’s expressive beliefs better predicted IPA (especially in women), and instrumental beliefs better predicted same-sex aggression (SSA). Primary psychopathy (involving the absence of anxiety) was associated with IPA for men, and with SSA in both sexes, whereas secondary psychopathy (involving lack of self-control) was associated with IPA and SSA in both sexes. In any case, in heterosexual relationships, the male aggressor and the female victim are much more common [20]. In their studies, antisocial traits (especially psychopathy) were the most predictive, and this occurred mainly in males. We also emphasize that psychopathy distinguishes offenders who have committed “common” crimes from those who have committed “blood” crimes, and some variables adequately predict whether those accused of blood crimes are more likely to attack their partners [21].

We highlight another peculiarity concerning the concept of psychopathy itself. There is an inverse relationship between the level of psychopathy and personality disorder, on the one hand, and psychotic alteration on the other [14]. Besides, studies on psychopathy show that Factor 2 (lifestyle/antisocial dimension) of Hare’s Psychopathy Checklist-Revised (PCL-R) was higher among homicidal men, whereas women tended to score higher on Factor 1 (interpersonal/affective dimension).

However, it is not our intention to focus on the biological basis of psychopathy and its possible relationship with IPA. We refer the reader to works on intimate partner aggression perpetration and how it corresponds to a dorsal-ventral gradient in medial PFC reactivity to interpersonal provocation [22], or that about the cardiac autonomic function [23], or that using fMRI technology [24], that about thyroid hormones [25], or that on head injuries [26].

Nor is it our intention to address the relationship between psychopathy, IPA, and drugs. This is a broad field, with representative works [12,27,28,29], which will not be addressed in this research.

### 1.3. Dark Personality

Subclinical psychopathy is one of the components of the so-called dark personality, along with variables such as Machiavellianism, subclinical narcissism, sadism, or moral disengagement [5]. An increasing number of works are linking the performance of IPA with the dark personality, both in a real and virtual form. In the latter case, there is a review which showed that the dark personality is related to cyberbullying, sending explicit unsolicited images, the non-consensual dissemination of ‘sexts’, sexual violence, or infidelity facilitated by technology [13]. These authors highlighted that within the dark personality, psychopathy is the trait most strongly associated with these online behaviors, with narcissism being the least closely related. Other studies reported that cyberstalking of previous and current intimate partners accounts for the most frequently reported harassment situations, which are continuously increasing and cause high levels of anxiety in the harassed victims [30]. These authors found that all the traits of the dark tetrad are significant predictors of the cyberstalking of the intimate partner.

A high level of subclinical psychopathy predicts most of the types of aggression that people can commit [18] and, in general, males score higher on all the scales of the dark personality. Aggressors of intimate partners also systematically score higher on all the scales, but again, essentially on psychopathy [31], and this is true for all types of aggression, whether physical, psychological, or sexual. A study showed that aggression increased if the couple had been in the relationship for a long time [32]. It should be noted that, in couples in which one of the members has dark personality traits and the relationship has existed for a long time, coercive sexual behaviors are quite likely to occur [33].

Regarding the relationship between dark personality and IPA, some researchers have proposed the creation of variables or constructs that explain social phenomena of interest related to violence, or links with some pathologies. Some are presented below:

- Dehumanization [16]: this theoretical concept states that psychopathy is associated with a variety of negative attitudes and behaviors towards women. For these authors, dehumanization is a mechanism that enhances the association between subclinical psychopathy and negative attitudes towards women. They argue that people with high psychopathic traits consider women as inhuman, and this dehumanizing assessment facilitates attitudes and behaviors that are consistent with the idea that women deserve to be treated like animals. The authors showed how psychopathy was indirectly related to sexist and violent attitudes towards women, with dehumanization being an important modulating variable.
- Attention deficit hyperactivity disorder (ADHD): there is a relationship between the symptomatology of ADHD and the perpetration of IPA and victimization of one’s partner. A study has studied this relationship and has also linked it to the abuse of alcohol and other drugs [34].
- Guilt and shame: the so-called “dark attitudes” have also been linked to IPA and psychopathy. One study have shown that shame moderates the association between a lack of social skills and perpetration of IPA, such that as shame increases, the relationship between lack of social skills and perpetration of IPA increases [35].
- Anger: in this case, studies have shown its relationship with IPA, but not with psychopathy because in primary psychopathy, individuals assault, but without feeling any anger [36].
- Animal abuse: Studies demonstrated that acts of IPA and animal abuse are phenomena that normally occur concurrently. It was also shown that animal abuse is habitually performed more by males than by females [37].
- Love: Some studies have linked the typologies of love (specifically, Sternberg’s model) to IPA and psychopathy. Studies corroborated the existence of significant negative relationships between pettiness and the love components of Sternberg’s model (intimacy, passion, and commitment) [15]. These authors also found that deficits in love explained the increase in aggression towards the partner.
- Revenge: sometimes one parent (usually the father) is capable of abusing the parent who has broken off the relationship by inflicting pain on their children [5,38].

We now refer to the procedures for the measurement of psychopathy. Self-reporting measures, so common in such studies, present the problem of subjects’ social desirability [10]. To avoid this phenomenon, one possibility is the use of implicit measures, and the Implicit Association Test (IAT) has been used effectively [39]. The only other alternative is the interview, as used in tests such as those created by Hare.

### 1.4. Typology

Reference to psychopathy in general should be nuanced. Many studies show that it is necessary to establish the dimension of psychopathy to which one refers. Depending on this, different typologies can be created. Thus, Iyican and Babcock, conceived of psychopathy as a personality disorder that has to do with antisocial, impulsive, and violent behavior, which necessarily includes IPA [40]. According to these authors, using the Psychopathic Personality Inventory, the Fearlessness-Dominance Factor I (PPI-I) evaluates the affective-interpersonal traits of psychopathy, whereas the Impulsive-Antisociality Factor II (PPI-II) evaluates the behavioral traits of the psychopath lifestyle. The data verify that all forms of violence correlate with IPP-I, such that people with high scores in the PPI-I dimension show an increased risk of IPA perpetration compared to those with high scores in the PPI-II.

Other typologies, for example, which establishes three types of aggressors depending on the level of violence and the aggressors’ degree of psychopathology: low (65%), moderate (27.8%), and high (7.1%) [17,41,42,43].

In some countries such as Spain, IPA has been referred to as gender-based violence. Although all IPV is considered gender-based violence, not all gender-based violence is IPV. In this paper, hereafter, we refer to IPV. Gender-based violence is an expression that has been consolidated to refer to the physical, psychological, sexual, or moral abuse that men exert against women and whose social entity has merited legislation and a specific line of procedural and criminal law (Organic Law 1/2004 of 28/December). The United Nations (UN, 1995) considers gender-based violence to be any sexist act that physically, psychologically, or sexually harms a woman just for being female, including threats and coercion, whether in public or in private. The World Health Organization (WHO, 2002) considers gender-based violence a major public health problem and a violation of human rights.

The following objectives were proposed in this study:

1. To determine whether Hare’s proposed psychopathy characteristics are maintained in a sample of inmates condemned for IPA.
2. To determine the most common psychopathy traits due to IPA among prisoners who are serving sentences under the tutelage of the Criminal Justice.
3. To determine which features of psychopathy proposed by Hare predict involvement in IPA.

## 2. Materials and Methods

### 2.1. Participants

This study included 92 men convicted of IPA serving their sentences in a regular prison in Córdoba (Spain). The inmates’ ages ranged from 22 to 61 years (*M* = 40.33, *SD* = 9.95), with convictions for IPA of between 4 and 405 months. Their time in prison ranged from one month to 15 years and three months. Of them, 30 were convicted exclusively for IPA crimes, and the rest had committed other crimes besides IPA. Similarly, 83 individuals had not committed homicide, 6 were convicted of attempted homicide, and 3 were convicted of homicide.

### 2.2. Instruments

Despite the limitations of this instrument [44], because of its ease of application and the possibilities for researchers to access the population under study, the Psychopathy Check- List (PCL-R) has been used [3], specifically, the revised Spanish version [45,46]. The PCL-R is a rating scale consisting of a semi-structured interview administered by experts. The scale consists of 30 items that are answered on a three-point Likert format: a score of zero indicates that the element does not apply to the evaluated individual; a score of one indicates that the element applies to some extent to the individual; and a score of two is general evidence that the element applies to many areas of an individual’s life. The maximum score is 60 points, and a score of 30 or more is the recommended limit for a psychopathy diagnosis, although a score higher than 20 is considered significant [47]. The PCL-R also provides a dimensional assessment of psychopathic traits [48], with two factors: Factor 1 (Interpersonal/Affective) and Factor 2 (Social Deviation), and four facets: Facet 1 (Interpersonal: Glibness/Superficial Charm, Grandiose Estimation of Self, Pathological Lying and Manipulativeness); Facet 2 (Affective: Lack of Remorse or Guilt, Shallow Affect, Callousness/Lack of Empathy, and Failure to Accept Responsibility for One’s Actions); Facet 3 (Lifestyle: Need for Stimulation, Parasitic Lifestyle, Lack of Realistic Long-Term Goals, Impulsivity, and Irresponsibility); and Facet 4 (Antisocial: Poor Behavioral Controls, Early Behavior Problems, Juvenile Delinquency, Revocation of Parole, and Criminal Versatility).

### 2.3. Design and Procedure

The research design used was ex post facto, cross-sectional with a single group, conducted through structured interviews [49]. The adequacy of this procedure is justified both by the living conditions of the persons interviewed and by the nature of the investigation.

Having defined the general framework for data collection, the general objectives, and the specific objective, we requested permission from the relevant Dean Judge, the higher judicial authorities, and the prisons.

Of the 242 inmates convicted for IPA, 92 agreed to participate and signed the informed consent. After the authorization processes were completed, including access to the prison records of inmates who had agreed to collaborate in the investigation, the interviews began in June 2016 and concluded in June 2017. All interviews were conducted individually with only the researcher and the interviewee, behind closed doors, and they lasted between 30 and 60 min.

### 2.4. Data Analysis

The metric study of the items and the dimensionality of the scale was performed through exploratory factorial analysis (EFA), using the Hull method [50] with direct oblimin rotation, which is appropriate when the correlation between the factors analyzed is known or assumed [51]. The adequacy of EFA on the matrix was tested with the Kaiser–Meyer–Olkin (KMO) test and Bartlett’s sphericity test. To analyze the discrimination of items, we applied the analysis of the item response theory, specifically the multidimensional discrimination index [52].

Subsequently, a confirmatory factor analysis (CFA) was carried out, which was estimated using the Least Square Robust method, appropriate to the categorical nature of the variables under study [53]. The fit of the models has been tested with the following indices: scaled Satorra–Bentler chi-square (χ2S-B) [54]; the comparative fit index (CFI) and the non-normality fit index (NNFI) (≥0.90 is adequate; ≥0.95 is optimal); the root mean square error of approximation (RMSEA) and the root mean square residual (SRMR) (≤0.08 is adequate; ≤ 0.05 is optimal) [55]. The developed in EQS 6.3.

Basic descriptive analyses of mean, standard deviation, normality, and kurtosis of the target variables were performed. A simple linear regression was performed to determine which psychopathy variables predict involvement in crimes of IPV and other crimes added to the convictions.

## 3. Results

We calculated the reliability of the instrument, obtaining the following results: alpha index of α = 0.82 and McDonald’s Ω = 0.92, which are acceptable, and in line with most studies using this instrument [46].

EFA was carried out to determine the grouping of the items. The results showed a Mardia multivariate kurtosis coefficient of 496.36. Bartlett’s statistic was χ^2^(190) = 728.3, *p* = 0.00, and the KMO test was 0.700. The factorial solution showed the adequacy of 6 factors, which explained 66.1% of the accumulated variance: F1 = 24.5%; F2 = 12.4%; F3 = 8.7%; F4 = 8.4%; F5 = 6.6%; and F6 = 5.2%. The fit indexes provided by the Hull method used show optimal values of CFI = 0.95, GFI = 0.99. Communality and factorial weights (see Table 1), as well as the discriminant multidimensionality index [52], whose values were greater than 0.20, indicated item discrimination. The factorial solution, which is presented below, did not match the factors or facets proposed by Hare.

EFA detected six factors with loadings higher than 5%. The first factor consisted of three items: Juvenile Delinquency, Early Behavior Problems, Criminal Versatility, and, to a lesser extent, Parasitic Lifestyle. Therefore, there was a general reference to the individual’s criminal history, coupled with having learned to perpetrate many criminal activities and being used to living off others. This factor could be called “Criminal Past and Criminal Knowledge”.

The second factor explained 12.4% of the total variance and was composed of the following items: Poor Behavioral Control, Impulsivity, and Superficial and Shallow Affect. Although the third aspect was somewhat different, the factor could be called “Impulsivity.”

The third factor explained 8.7% of the variance and included the items Glibness/Superficial Charm, Grandiose Estimation of Self, and Need for Stimulation/Tendency to Boredom. Again, the third element was somewhat discrepant, but the factor could be called the “Need to stand out.”

The fourth factor explained 8.4% of the variance and consisted of two items: Lack of Remorse or Guilt and Failure to Accept Responsibility for One’s Actions. It could be called “Lack of Empathy.”

The fifth factor explained 6.6% of the variance and comprised the items Pathological Lying, Swindler/Manipulativeness, Irresponsibility, and, to a lesser extent, Affective Insensitivity/Lack of Empathy. The score of this last item of this factor was not problematic despite there already existing a factor that we have called “lack of empathy”, as this factor refers to “Manipulation of others.”

Finally, the sixth factor, that barely exceeded explaining 5% (5.2%) of the variance, comprised two items: Many short-term marital relationships and Sexual promiscuity. We called it “Instability in couple relationships.” We note that the first factors loaded higher than the next ones, due to the variance extraction method, so this should be taken into account when analyzing these results.

**Figure 1:**
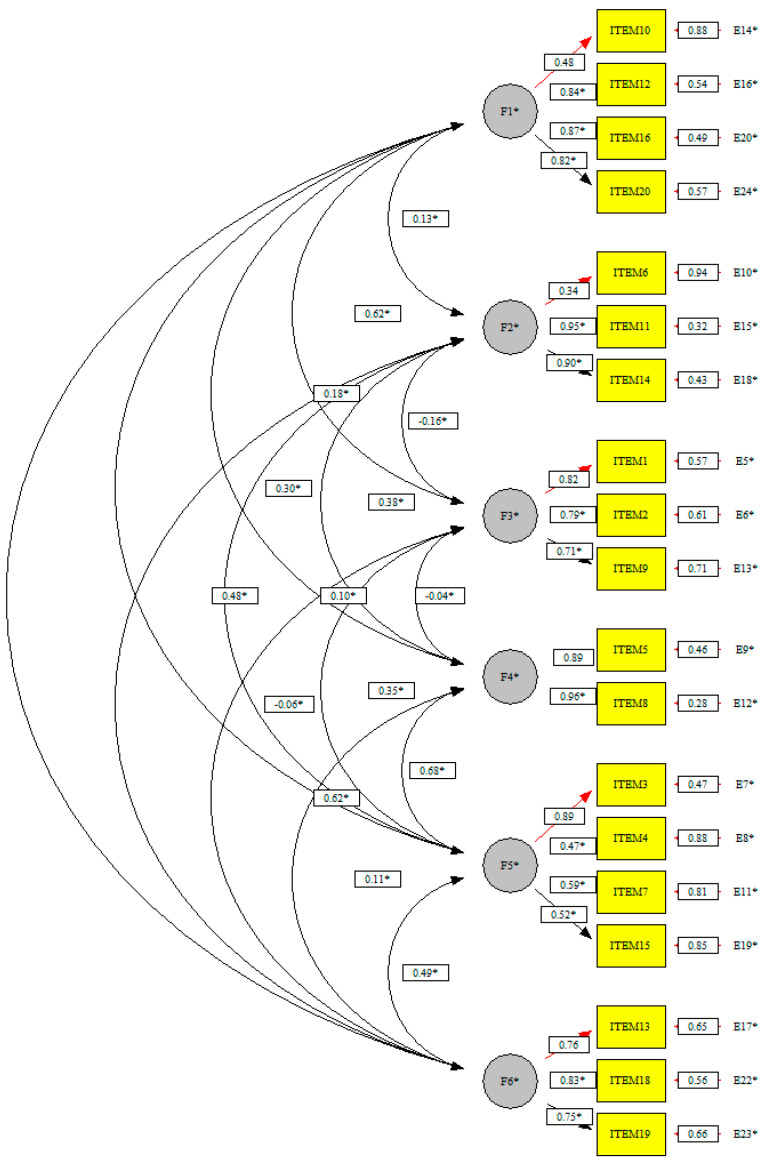
Graphical solution of CFA.

Based on the results obtained in the EFA, the CFA has been developed with optimal results: χ^2^ S-B = 115.15; *p* = 0.30; RMSEA = 0.06; SRMR = 0.09; CFI = 0.99; NNFI = 0.99 (see Figure 1).

Subsequently, descriptive analyses of the target variables, individually and grouped by the instrument’s previously found factorial solution, were carried out (see Table 2). In this regard, the impulsivity item showed the highest mean. As for the factorial solution, Factor 4 (Lack of empathy) obtained the highest mean scores.

Subsequently, concerning our second objective, following the recommendations of Hare [56,57], the individuals who had a psychopathy diagnosis with scores > 30 were selected, finding *n* = 2 (2.2% of the total sample). When following the criterion of a score above 20 indicating that psychopathic traits are considered significant [47], the number of subjects increased to *n* = 26 (28.3% of the total sample).

In accordance with our third objective, two linear regression models were calculated, with the perpetration of IPA as the dependent variable. The first model was performed with the individual Hare questionnaire items and the following sociodemographic variables: age, months of prison sentence, and attempted or committed homicide. The model, *F*(4, 91) = 27.32, *p* < 0.001; Adjusted *R*^2^ = 0.53; Durbin–Watson statistic = 1.86, included four variables, with the most important for the prediction of the crimes of IPA being criminal versatility (see Table 3). The linear regression performed as a function of the prediction of psychopathy revealed that the variables criminal versatility, as well as poor behavioral controls, swindling/manipulating, and the number of months of time served were all positively significant. This result is consistent with the results of the EFA.

The second model included the factors found in the previously performed EFA, with the same variables: age, months of prison sentence, and attempted or committed homicide. The model, *F*(4, 91) = 17.84, *p* < 0.001; Adjusted *R*^2^ = 0.42.; Durbin–Watson statistic = 1.92, included four predictor variables, the most important being Factor 1 (see Table 4). In the regression performed, we entered the new factors found by EFA in the equation: Factor 1 (Criminal past and criminal record) and Factor 2 (Impulsivity) and included two more variables: prison sentence in months and having committed homicide. The result confirmed that the factors found were valid.

## 4. Discussion

The study did not confirm the factorial configuration reported by Hare, but it detected six factors, in our view, that were much more concrete and clear and, in this case, applied to males who had assaulted their intimate partners. These six factors are: Criminal record, Impulsivity, Need to stand out (which could also be defined as narcissism), Lack of empathy, Manipulation of others, and Instability in couple relationships. The last factors explain less variance than the first ones, but their characterization is typical of psychopathy. Therefore, we can state that males who assault their partner, who have attempted to murder them or have consummated the homicide have a clearly psychopathic profile. Also, the first two factors are high predictors of psychopathy, according to Hare’s evaluation procedure.

Our results match those of the main comprehensive reviews in this regard, which consider the results as typical of the relationship between psychopathy and IPA. Specifically, one study includes quite a few investigations that relate to the Factor 1 we detected [8], and, there are references to works that address the six factors, especially the sixth, the lack of a stable partner [6]. This topic of the partner is also reflected in one work among others [11].

On another hand, it is noteworthy that the factors detected are usually present in the dark personality (the lack of empathy in all its types, the manipulation of others, the desire to be the center of attention, etc.), so our results coincide with those of reviews and authors already mentioned in the introduction of this work [18,31,32,33].

The concept of psychopathy predates that of the Dark Personality. This last concept encompasses one of the dimensions of psychopathy, the so-called subclinical. However, there are studies that show that the various differences within psychopathy are not well defined, and that, for example, between secondary and subclinical psychopathy there is no clear distinction. On the other hand, within Social Psychology the concept of Machiavellianism was created, which has very high correlations with the so-called subclinical psychopathy and with subclinical narcissism. This work has shown how existing studies in the literature on the subject show that there are more coincidences than differences between the three cited concepts. Specifically, with regard to intimate couple relationships, studies agree that aggressors present high levels of the variables that comprise the Dark Personality (including sadism). In this sample, information has been collected from participants who have been convicted by law of attempted murder or the murder of their partner. The data shown here allows us to re-define Hare’s work, in a way that simplifies his system, more intuitive than scientific. This will allow, in future research, to study the relationship between the new factors found and the dimensions of the dark personality.

This work will prevent cases of violence against the partner, through the creation of preventive programs carried out by community social services. The social services and the police are the institutions that usually have knowledge of IPA cases, and must act by locating the alleged aggressor and verifying if he presents the psychopathy factors detected here, so that it can intervene to prevent a woman from suffering the consequences of being attacked. We consider, therefore, that this work has a high social impact.

## 5. Conclusions

This study presents some limitations, such as having worked only with males, not having a control sample, or a sample of inmates not diagnosed with psychopathy or who had not committed IPA. These limitations could not be overcome due to the difficulty of accessing incarcerated subjects who are serving sentences for IPA. However, precisely this limitation is the strong point of this work: a broad sample of people who had assaulted their intimate partners, including some who had murdered them.

Another limitation is that the sample only includes males, although almost all the works performed on this topic agree that, in the case of serious aggressions and even death within the couple, the aggressor is the male [14].

However, it also has the positive value of having been made with samples of people tried and convicted of having attempted against their partner, and who have also been diagnosed as psychopaths. Precisely this advantage has been accompanied by the impossibility of applying psychometric tests, due to the limitations implied by access to inmates within the prison.

Future investigations should detect some variables in partner aggressors that are more focused on why such violent acts are committed (in that sense, the works focusing on the concept of revenge can be of great interest [5,38,58]), as well as the existence of explanatory mediating variables.

## Figures and Tables

**Table 1:** Factorial Solution of the PLC-R.

Items	F1	F2	F3	F4	F5	F6	Com.	MDISC
1. Glibness/Superficial charm			0.84				0.79	1.88
2. Excessive sense of self-worth			0.63				0.57	1.04
3. Pathological lying					0.62		0.66	1.25
4. Swindler/Manipulator					0.54		0.48	0.98
5. Lack of remorse or guilt				0.85			0.73	1.66
6. Superficial and shallow affection		0.31					0.37	0.44
7. Affective insensitivity/lack of empathy					0.32		0.30	0.56
8. Inability to accept responsibility for one’s actions				0.82			0.72	1.59
9. Need for stimulation/tendency to boredom			0.44				0.65	1.12
10. Parasitic lifestyle	0.34						0.30	0.46
11. Poor behavioral controls		0.82					0.64	1.36
12. Early behavior problems	0.75						0.65	1.33
13. Lack of realistic long-term goals						0.53	0.55	1.06
14. Impulsivity		0.70					0.51	1.02
15. Irresponsibility					0.37		0.36	0.52
16. Juvenile Delinquency	0.95						0.94	4.07
17. Revocation of parole							0.08	0.33
18. Sexual promiscuity						0.64	0.56	1.01
19. Frequent brief marital relationships						0.90	0.80	2.09
20. Criminal versatility	0.65						0.47	0.92

Note: Com.: Communality; MDISC: Multidimensional discriminant index.

**Table 2:** Descriptive statistics of the target variables.

Factors and Items	M	SD	Asymmetry	Kurtosis
Total PCL-R	16.84	6.06	0.55	0.9
F1: Criminal past and criminal record	0.64	0.60	0.53	−1.01
F2: Impulsivity	1.65	0.34	−1.20	1.40
F3: Need to stand out	0.41	0.58	1.43	1.15
F4: Lack of empathy	1.70	0.52	−1.63	1.64
F5: Manipulation of others	0.81	0.38	0.63	0.25
F6: Instability in couple relationships	0.45	0.57	1.01	−0.12
Glibness/Superficial charm	0.45	0.701	1.27	0.22
Grandios estimation of self	0.39	0.628	1.37	0.77
Pathological lying	0.67	0.613	0.32	−0.62
Swindler/Manipulator	0.23	0.494	2.11	3.81
Lack of remorse or guilt	1.71	0.584	−1.87	2.45
Superficial and shallow affection	1.14	0.720	−0.21	−1.03
Affective insensitivity/lack of empathy	1.14	0.526	0.16	0.39
Failure to accept responsibility for one’s actions	1.70	0.550	−1.64	1.83
Need for stimulation/tendency to boredom	0.41	0.729	1.44	0.47
Parasitic lifestyle	0.14	0.408	3.02	9.07
Poor behavioral controls	1.90	0.365	−4.01	16.42
Early behavior problems	0.83	0.833	0.33	−1.48
Lack of realistic long-term goals	0.38	0.552	1.09	0.21
Impulsivity	1.91	0.320	−3.95	16.62
Irresponsibility	1.22	0.590	−0.08	−0.36
Juvenile delinquency	0.64	0.833	0.76	−1.12
Revocation of parole	0.03	0.179	5.35	27.22
Sexual promiscuity	0.64	0.884	0.777	−1.27
Frequent brief marital relationships	0.34	0.616	1.659	1.59
Criminal versatility	0.97	0.919	0.065	−1.83

Note: M: Mean; SD: Standard Deviation.

**Table 3:** Linear regression indices of the first model.

Model	B	SE	Beta	t	Sign.	Tol.	VIF
Constant	1.593	0.499		3.193	0.002		
Criminal versatility	0.904	0.110	0.637	8.241	0.000	0.852	1.174
Months of prison sentence	0.004	0.001	0.231	2.992	0.004	0.858	1.166
Poor behavioral controls	0.600	0.257	0.168	2.333	0.022	0.983	1.017
Swindler/Manipulator	0.413	0.190	0.157	2.172	0.033	0.981	1.020

Note: B: Beta; SE: Standard error; Sign.: significance; Tol.: Tolerance; VIF: Variance inflation factor.

**Table 4:** Linear regression indices with Factorial Confirmatory Analysis factors.

Model	B	SE	Beta	t	Sign.	Tol.	VIF
Constant	1.688	0.513		3.292	0.001		
F1: Criminal record	1.067	0.183	0.499	5.822	0.000	0.861	1.161
Months of prison sentence	0.007	0.002	0.402	4.243	0.000	0.702	1.424
F2: Impulsivity	0.780	0.307	0.209	2.542	0.013	0.936	1.068
Committed homicide	0.670	0.284	0.218	2.363	0.020	0.739	1.353

Note: B: Beta; SE: Standard error; Sign.: significance; Tol.: Tolerance; VIF: Variance inflation factor.

## Data Availability

The data presented in this study are available on request from the corresponding author. The data are not publicly available due to restrictions from the public administration.
